# Trends in the Intake of Energy, 2015–2024 - KNHANES

**DOI:** 10.56786/PHWR.2026.19.38.4

**Published:** 2026-10-08

**Authors:** Jinyoung Park

**Affiliations:** Division of Health and Nutrition Survey and Analysis, Department of Chronic Disease Prevention and Control, Korea Disease Control and Prevention Agency

Energy intake among those aged 1 and over was 2,136 kcal for males and 1,580 kcal for females in 2024, which has decreased over the past 10 years. The percentage of energy intake from fat has increased, while the percentage from carbohydrates has decreased (Figure 1).

## Supplementary Material



## Figures and Tables

**Figure 1:**
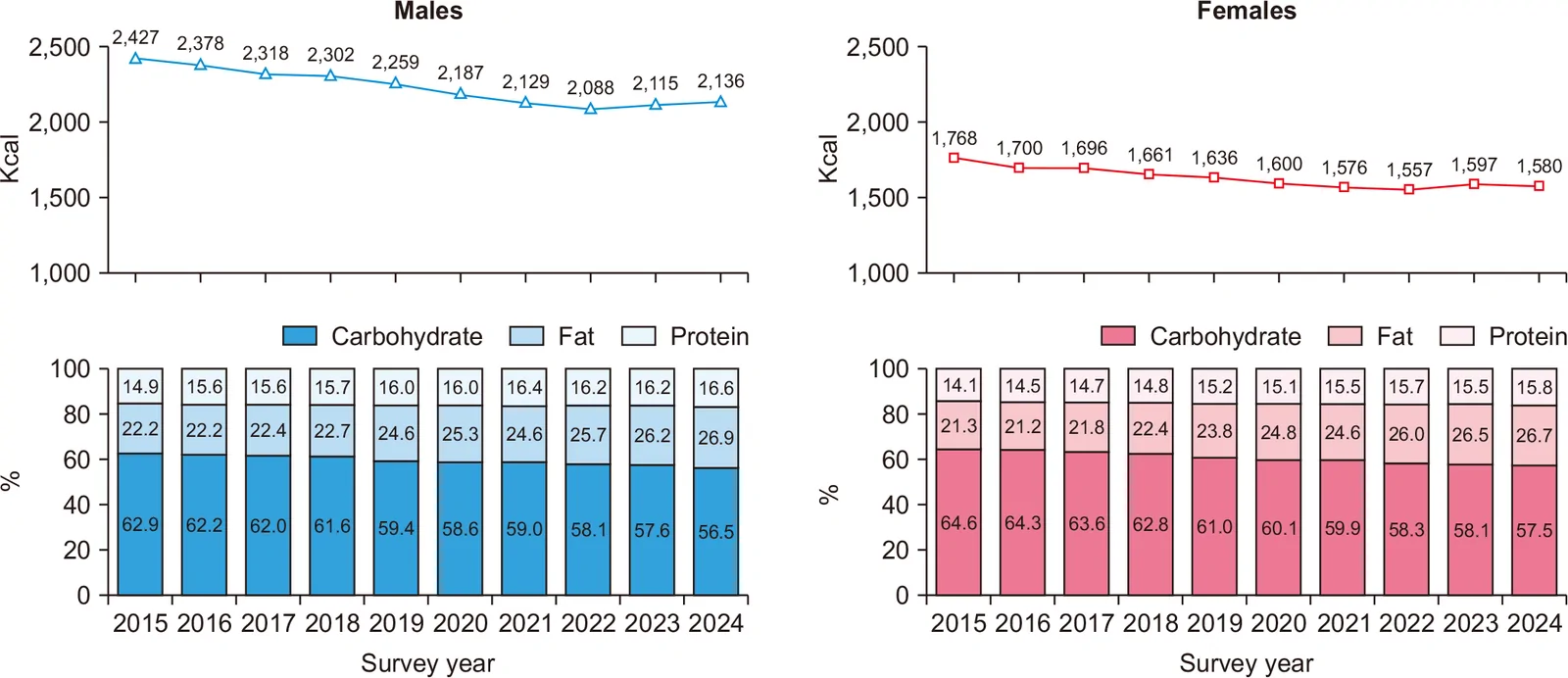
Trends in energy intake and percentage of energy intake from carbohydrate, fat, and protein *Intake of Energy: mean value per person per day, among those aged ≥1 years. **Energy intake ratios of protein, fat, and carbohydrates: the proportion of (protein intake)×4, (fat intake)×9, or (carbohydrate intake)×4 relative to {(protein intake)×4+(fat intake)×9+(carbohydrate intake)×4}, among those aged ≥1 years. ※The mean value in Figure 1 was calculated using the 2005 Population Projections for Korea.

