# Supplementary material for: Trends in the Intake of Energy, 2015–2024 - KNHANES
Source: Public Health Wkly Rep. 2026 Oct 8;19(38):1648–9. doi: 10.56786/PHWR.2026.19.38.4 (PMC13654657; doi:10.56786/PHWR.2026.19.38.4)
Supplement: Translation

# Translation: 에너지 섭취량 추이, 2015–2024년 - 국민건강영양조사

**Authors:** 진영 박

**Affiliations:** 질병관리청 만성질환관리국 건강영양조사분석과

최근 10년간 에너지 섭취량(1세 이상)은 감소 경향으로 2024년 기준 남자 2,136 kcal, 여자 1,580 kcal였다. 지방을 통한 에너지 섭취분율은 증가한 반면, 탄수화물을 통한 에너지 섭취분율은 감소하였다(그림 1).

## Figures and Tables

**그림 1:** 에너지 섭취량 및 급원별 섭취분율 추이 *에너지 섭취량: 1인 1일당 에너지 섭취량의 평균, 1세 이상 **단백질‧지방‧탄수화물 에너지섭취분율: {(단백질 섭취량)×4+(지방 섭취량)×9+(탄수화물 섭취량)×4}에 대한 {(단백질 섭취량)×4} 또는 {(지방 섭취량)×9} 또는 {(탄수화물 섭취량)×4}의 분율, 1세 이상 ※그림 1의 섭취량 및 분율은 2005년 추계인구로 연령표준화
